# Association between serum antibodies against oral microorganisms and vitamin D deficiency among US adults: A cross-sectional study

**DOI:** 10.1097/MD.0000000000050348

**Published:** 2026-08-28

**Authors:** Li Tan, Si-qun Xu

**Affiliations:** a Department of Conservative and Endodontic Dentistry, Xiangya Stomatological Hospital and Xiangya School of Stomatology, Central South University, Changsha, China; b Hunan Key Laboratory of Oral Health Research, Central South University, Changsha, China; c Department of Oral and Maxillofacial Surgery, Second Xiangya Hospital of Central South University, Changsha, Hunan, China.

**Keywords:** antibody, NHANES III, oral microorganisms, serum 25-Hydroxyvitamin D, serum Antibodies

## Abstract

Vitamin D deficiency is common and may contribute to periodontal disease by affecting periodontal pathogens. Most studies analyzing this association have focused mainly on a specific periodontal bacterium (*P gingivalis*) and have involved relatively small study populations (tens or hundreds of individuals). To address this gap, a sizable, nationally representative adult population was utilized to investigate the association between the prevalence of Vitamin D deficiency and immunoglobulin G (IgG) antibodies to 19 periodontal bacteria using a large, nationally representative population. Drawing from the Third National Health and Nutrition Examination Survey, which constitutes a cross-sectional representation of the noninstitutionalized US population encompassing 33,994 individuals, we incorporated all qualifying participants aged 40 and above. These participants were then categorized into 2 groups according to serum 25-hydroxyvitamin D (25 [OH]D) concentration levels, following the Endocrine Society Clinical Practice Guidelines: Vitamin D deficiency (<75.0 nmol/L) and sufficient (≥75.0 nmol/L). We subsequently excluded individuals without complete records of serum IgG antibodies against periodontal bacteria, resulting in a final analytic sample of 6221 individuals. Using cluster analysis based on the Socransky classification scheme for oral microorganisms, the 19 antibody titers were divided into 4 clusters: Red-Green, Orange-Blue, Yellow-Orange, and Orange-Red. After controlling for lifestyle and demographic factors, no significant correlations were found between any of the clusters and Vitamin D deficiency. However, when analyzing each of the 19 periodontal serum IgG antibodies individually, only *P gingivalis* antibody levels remained positively associated with Vitamin D deficiency after full adjustment (odds ratio [OR] = 1.049; 95% confidence interval [CI]: 1.008–1.091; *P* < .05). In contrast, antibodies against *A actinomycetemcomitans* and *E nodatum* were inversely associated with Vitamin D deficiency (*A actinomycetemcomitans*: OR = 0.939; 95% CI: 0.895–0.986; *P* < .05; *E nodatum*: OR = 0.947; 95% CI: 0.911–0.985; *P* < .01). No other significant associations were observed after covariate adjustment. There were no associations between antibody clusters and vitamin D deficiency. However, specific bacteria (*P gingivalis*, *A actinomycetemcomitans*, and *E nodatum*) were associated with vitamin D status.

## 1. Introduction

Vitamin D is a fat-soluble hormone obtained mainly from sunlight, with additional sources from diet and supplements.^[1–3]^ It plays a crucial role in calcium-phosphate regulation and bone metabolism by enhancing calcium absorption, reducing parathyroid hormone secretion, and decreasing bone resorption.^[4,5]^ Vitamin D also stimulates bone production, optimizes bone remodeling, and increases bone matrix proteins.^[6,7]^ Due to the high prevalence of deficiency, insufficient vitamin D levels are linked to reduced bone mineral density, impaired bone healing, and bone metabolism disorders.^[8,9]^

The nutritional impact of vitamin D on oral and periodontal health has garnered increasing attention.^[10–12]^ Over the years, numerous studies have shown that individuals with vitamin D deficiency tend to have poorer periodontal health.^[8,13–15]^ Moreover, some in vitro studies suggest that vitamin D may reduce the number of *P gingivalis*, a key periodontal pathogen, through mechanisms such as active autophagy and the induction of antimicrobial peptide production,^[16–18]^ and could potentially alleviate the inflammatory burden of periodontitis in animal models.^[19–21]^ This indicates that vitamin D might regulate periodontal pathogens, linking it closely with the development of periodontal disease. However, comprehensive population-based studies investigating the relationship between vitamin D deficiency and a broader spectrum of periodontal pathogens remain limited.

To explore this relationship, serological markers such as immunoglobulin G (IgG) antibody titers can serve as useful indicators of historical and chronic exposure to specific periodontal pathogens. Even after periodontal treatment, IgG antibody titers produced upon infection might persist for up to almost 3 years.^[22]^ IgG antibody titers against *P gingivalis* and *A actinomycetemcomitans* in patients with periodontitis remain constant over 15 years.^[23]^ Therefore, IgG antibody titers against oral microorganisms may be a cumulative predictor of periodontal infection. The Centers for Disease Control and Prevention reported IgG antibody titer levels for oral bacteria in 2008^[24]^ after analyzing serum samples collected and stored from Third National Health and Nutrition Examination Survey (NHANES III) participants. Importantly, the 19 bacterial IgG antibodies have been previously categorized into 4 clusters based on their intercorrelations and associations with periodontal disease. We therefore analyzed both cluster scores and individual antibody levels: the former to capture potential synergistic effects of related bacterial groups, and the latter to identify pathogen-specific signals that may be masked when data are aggregated. Similarly, serum 25-hydroxyvitamin D (25[OH]D), the primary circulating form of vitamin D and a widely accepted biomarker for assessing vitamin D status, is also available in the NHANES III dataset. Based on these data, the current study intends to evaluate the relationship between serum antibody titers against 19 specific bacteria and the estimated prevalence of vitamin D deficiency in a sizable, nationally representative sample of adult U.S. citizens. We hypothesized that vitamin D deficiency would be positively associated with antibody levels against periodontal pathogens, particularly those in the Orange-Red cluster (e.g., *P gingivalis*), and that this association would persist after adjusting for demographic and lifestyle factors.

## 2. Methods

### 2.1. Source of data

The NHANES III data, a nationwide representative sample of noninstitutionalized U.S. adults gathered by multistage probability clustered sampling, was used in this study for analysis. Specifically, information from NHANES III, including serum 25(OH)D concentrations, clinical and antibody data related to periodontal disease, as well as various physical health risk factors and behaviors, was collected in this study.^[25]^

The study participants are NHANES III respondents who are 40 years of age or older and have complete blood IgG antibody data for 19 oral microorganisms. Participant records have been linked to the dental, medical, and socioeconomic status information from NHANES III. All of the data used in this study may be found at https://wwwn.cdc.gov/nchs/nhanes/nhanes3/default.aspx.

### 2.2. Research cohort

**Figure 1.**
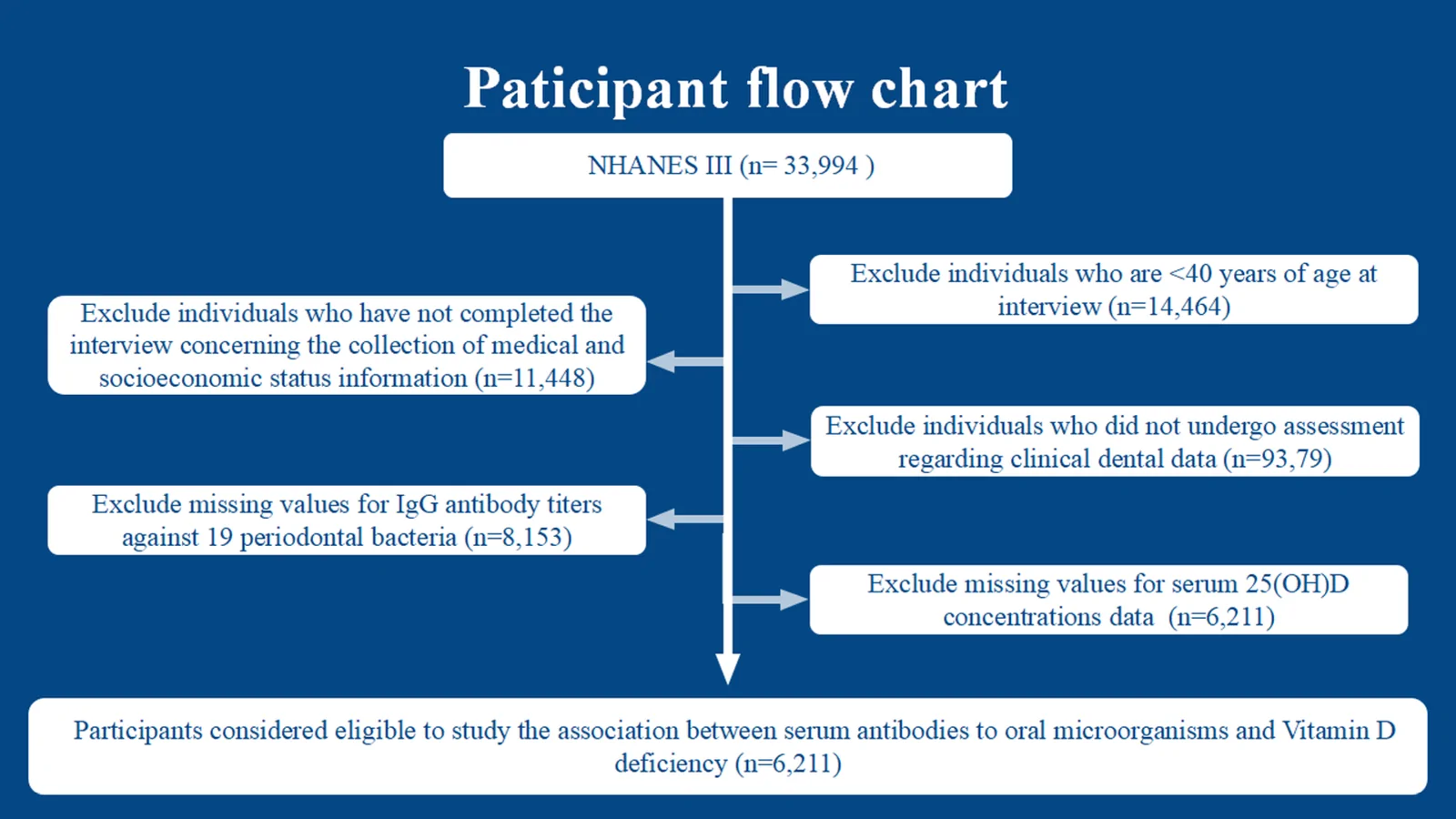
The flowcharts for the inclusion and exclusion of individuals in this study.

Among the 33,994 participants, 14,464 were aged 40 years or older. Of these, 11,448 completed the interview concerning the collection of medical and socioeconomic status information, 9379 underwent evaluation regarding clinical dental data, and 8153 provided serum samples for measuring IgG antibodies against 19 periodontal bacteria. After excluding individuals with missing serum 25(OH)D data, 6211 participants comprised the final analytic sample (Fig. 1).

### 2.3. Exposure measures

Using the “checkerboard” immunoassay technique, serum samples from 8153 NHANES III adults over 40 years of age were examined for IgG antibodies directed against 19 different oral bacterial species^[26]^; Additional methodological information is provided in the NHANES III documentation, and the data were quantified in gravimetric units.

Serum IgG antibody titer levels for each bacterial species were logarithmically transformed, and normalization was obtained by dividing by the log-transformed sample standard deviation. According to a prior study, 4 independently distinct groups of IgG antibodies against periodontal bacteria were found by incorporating normalized *z* scores of IgG antibodies against 19 of these bacteria into cluster analysis (The names of the 4 clusters: Orange-Red, Red-Green, Yellow-Orange, and Orange-Blue may indicate various stages of periodontal health).^[27]^ Cluster scores, which were the main exposure factors for this analysis, were calculated for each study participant by adding the standardized z scores of IgG titers against oral bacteria within each of the 4 clusters.

### 2.4. Outcome measures

The DiaSorin radioimmunoassay kit (Stillwater, MN) was used to test the serum 25(OH)D concentrations in the NHANES III (1988–1994). The regression approach was used to transform serum 25(OH)D data from NHANES III to equivalent 25(OH)D values from the liquid chromatography-tandem mass spectrometry method, and the details have been documented in a previous study.^[28]^ For all analyses, we used the liquid chromatography-tandem mass spectrometry-equivalent data, as advised by the Centers for Disease Control and Prevention.^[28]^

### 2.5. Covariate information

A wide range of demographic and socioeconomic characteristics were included in the covariates, which include age, sex, and race/ethnicity. In addition, low (≤6 years), mild (7–12 years), and high (≥13 years) educational attainment were taken into account, as was the income-to-poverty ratio. According to their smoking status, participants were further divided into 2 groups: smokers, who had smoked more than 100 cigarettes in their lifetime, and nonsmokers, who had smoked fewer than 100 cigarettes. In terms of alcohol intake, the participants were split into drinkers (those who drank more than 12 drinks annually) and nondrinkers (those who drank <12 drinks annually). Precise measures of height and weight were taken during the physical examination, and the results were used to calculate the body mass index (BMI).

### 2.6. Statistical analysis

Descriptive statistics were calculated using *t* tests and classification analysis methods, with careful consideration of the weights required by the complex survey design. According to the Endocrine Society Clinical Practice Guidelines, serum 25(OH)D concentrations were categorized into 2 groups: vitamin D deficiency (<75.0 nmol/L) and sufficient (≥75.0 nmol/L)^[29]^ (We acknowledge that alternative cutoffs such as <50 nmol/L exist in other guidelines. However, we selected the <75 nmol/L threshold for 2 main reasons: it offers a more conservative definition for capturing suboptimal vitamin D status relevant to chronic inflammatory conditions and using a lower cutoff would substantially reduce the deficient group size, potentially compromising statistical power for multivariable analyses). To investigate the relationship between the 4 *z*-score clusters and the presence of vitamin D deficiency, logistic regression models were applied. Additionally, the correlation between elevated levels of the 19 individual periodontal bacterial antibodies and the presence of vitamin D deficiency was examined. In particular, the association between vitamin D deficiency and antibody levels of the 19 specific periodontal microorganisms was evaluated using logistic regression models. These models were also used to investigate the association between the presence of vitamin D deficiency and clinical periodontal parameters, such as clinical attachment loss, probing depth, and the decayed, missing, and filled surfaces (DMFS) index. Age, sex, race, ethnicity, education, smoking status, alcohol consumption, BMI, and other possible confounding variables were carefully taken into account in all logistic regression models. Prior to logistic regression, we assessed multicollinearity among covariates using variance inflation factors. Variables with variance inflation factors >10 were considered to indicate significant collinearity and were evaluated for exclusion or combination to ensure model stability. For missing covariate data (including BMI, smoking, education, and income), complete-case analysis was applied, meaning that participants with missing values on any covariate were excluded from the multivariable models. All logistic regression analyses incorporated the complex sampling design of NHANES III, including sampling weights, strata, and primary sampling units, to ensure nationally representative estimates. R 4.1.0 and SPSS software were used for statistical analysis. The two-sided significance levels were established using predetermined thresholds of 0.05, 0.01, and 0.001.

### 2.7. Ethical approval

This study used de-identified, publicly available data from NHANES III. The original NHANES III protocols were approved by the National Center for Health Statistics Research Ethics Review Board, and all participants provided written informed consent. Our analysis of these de-identified data was exempt from additional institutional review board approval.

## 3. Results

### 3.1. Population characteristics of NHANES

6211 U.S. adults having relevant data on 19 periodontal blood IgG antibodies and serum 25(OH)D concentrations were eligible for investigation during the NHANES III study period. A thorough overview of these individuals’ main features is provided in Table 1. According to the Endocrine Society Clinical Practice Guidelines, 5146 (82.3%) of them satisfied the diagnostic requirements for vitamin D deficiency. Those with a deficiency were more likely to be female, non-Hispanic Black, Mexican American, nonsmokers or nondrinkers, and obese, and they were also typically younger than those with adequate serum 25(OH)D levels. They also tended to have worse oral and periodontal health, lower household incomes, and lower levels of education.

**Table 1:** Baseline characteristics of the study population in NHANES III and prevalence of vitamin D deficiency (weighted) by characteristics.

Characteristics	Overall (n = 6211)	Vitamin D deficiency (n = 5146)	Vitamin D sufficiency (n = 1065)	*P*-Value
Age, yr	55.98 ± 10.57	55.66 ± 10.55	57.65 ± 10.51	<.001
Gender				
Male	2888 (46.5%)	2270 (44.1%)	618 (58.0%)	<.001
Female	3323 (53.5%)	2876 (55.9%)	447 (42.0%)	
Ethnicity				
Non-Hispanic White	2747 (44.2%)	2003 (38.9%)	744 (69.9%)	<.001
Non-Hispanic Black	1608 (25.9%)	1501 (29.2%)	107 (10.0%)	
Mexican American	1583 (25.5%)	1406 (27.3%)	177 (16.6%)	
Other Race	273 (4.4%)	236 (4.6%)	37 (3.5%)	
Education, yr				
≤6	1091 (17.7%)	923 (18.1%)	168 (15.8%)	<.001
7–12	3421 (55.4%)	2857 (55.9%)	564 (53.2%)	
≥13	1660 (26.9%)	1331 (26.0%)	329 (31.0%)	
Family income-to-poverty ratio	2.69 ± 1.90	2.61 ± 1.86	3.10 ± 2.03	<.001
BMI(kg/m2)	27.34 ± 5.29	27.61 ± 5.48	26.10 ± 4.14	<.001
Smoking status				
nonsmoker	2672 (43.0%)	2279 (44.3%)	393 (36.9%)	<.001
Smoker	3539 (57.0%)	2867 (55.7%)	672 (63.1%)	
Alcohol consumption				
nondrinker	3435 (55.4%)	2892 (56.3%)	543 (51.1%)	<.001
Drinker	2761 (44.6%)	2242 (43.7%)	519 (48.9%)	
Mean attachment loss (mm)	1.68 ± 1.39	1.70 ± 1.41	1.58 ± 1.34	<.001
Percentage of sites with attachment loss ≥ 5mm (%)	7.47 ± 17.26	7.60 ± 17.37	6.77 ± 16.69	<.001
Mean probing depth (mm)	1.61 ± 0.62	1.63 ± 0.62	1.52 ± 0.61	<.001
Percentage of probing depths ≥ 4mm (%)	3.56 ± 9.50	3.71 ± 9.66	2.83 ± 8.62	<.001
DMFT	16.78 ± 7.92	16.58 ± 7.92	17.76 ± 7.85	<.001

All estimates accounted for complex survey designs.

BMI = body mass index, DMFT = decay, miss, fill index, NHANES = National Health and Nutrition Examination Survey.

### 3.2. Association between periodontal bacteria cluster scores and Vitamin D deficiency

**Figure 2.**
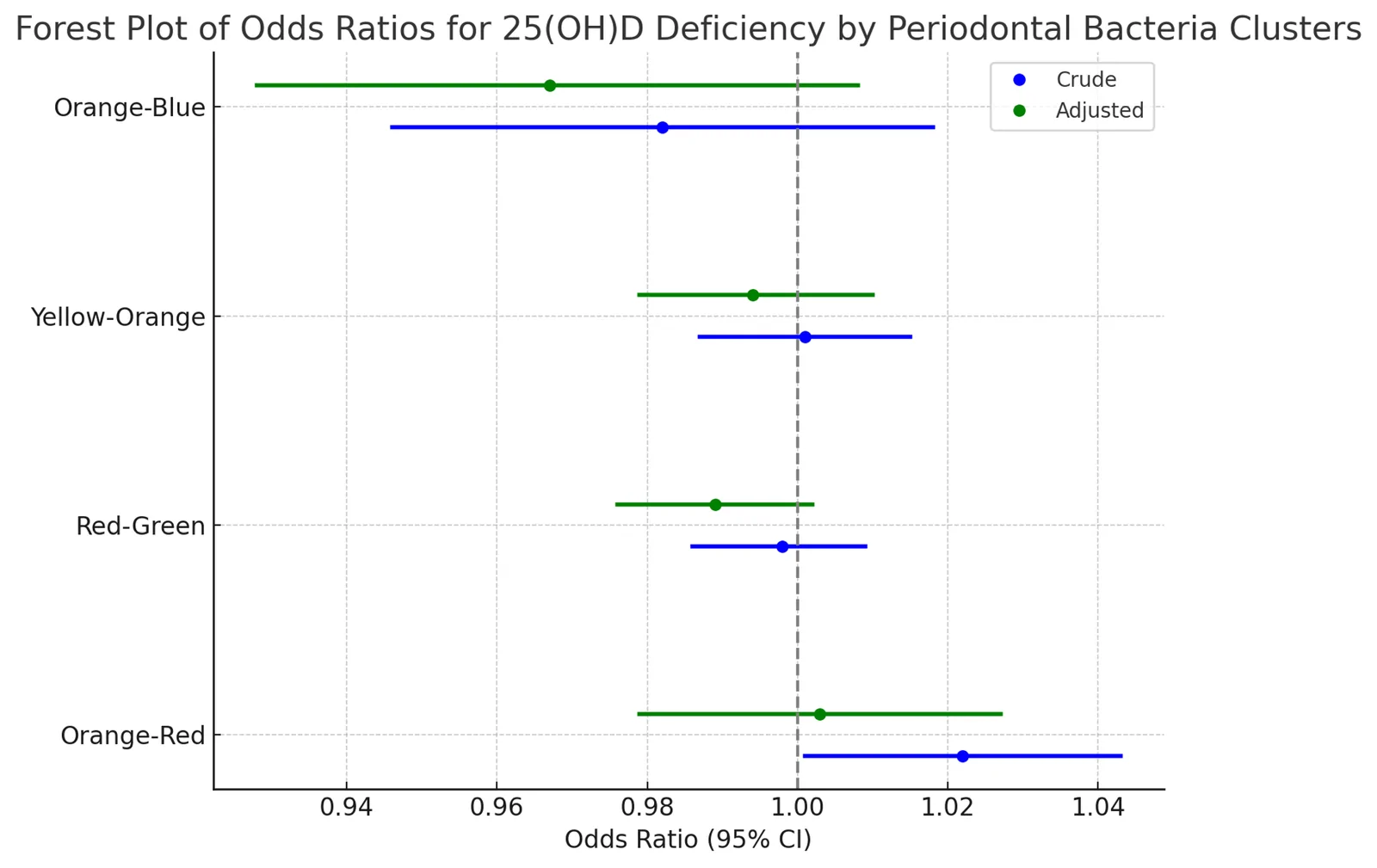
Forest plot of odds ratios (OR) and 95% confidence intervals (CI) for prevalent serum 25-hydroxyvitamin D (25[OH]D) deficiency according to periodontal bacteria cluster scores among participants in NHANES III. NHANES = Third National Health and Nutrition Examination Survey.

The prevalence of vitamin D deficiency increased by 2.2% for every unit rise in the Orange-Red cluster score, when covariates were not taken into account (odds ratio [OR] (95% confidence interval (CI) = 1.022 [1.001, 1.043]; *P* < .05) (Fig. 2) (Table 2). In contrast, the crude model did not show any significant connections for the Orange-Blue, Yellow-Orange, or Red-Green clusters (*P* > .05 for all) (Fig. 2) (Table 2). None of the 4 antibody clusters, however, showed any significant correlations with the prevalence of vitamin D deficiency after adjusting for age, sex, education, smoking status, alcohol consumption, and BMI (all *P*-values > .05) (Fig. 2) (Table 2).

**Table 2:** Odds ratio (95% CI) for prevalent vitamin D deficiency according to periodontal bacteria cluster scores among participants in the third National Health and Nutrition Examination Survey (NHANES III), 1988–1992. (N = 6211).

Periodontal bacteria antibody clusters	Crude Vitamin D Deficiency (5146/6211)	*P*-value	Adjusted* Vitamin D deficiency (5146/6211)	*P*-value
Orange-Blue†	0.982 (0.946, 1.018)	.32	0.967 (0.928, 1.008)	.11
Yellow-Orange‡	1.001 (0.987, 1.015)	.92	0.994 (0.979, 1.010)	.47
Red-Green§	0.998 (0.986, 1.009)	.68	0.989 (0.976, 1.002)	.91
Orange-Red‖	1.022 (1.001, 1.043)	<.05	1.003 (0.979, 1.027)	.82

The clusters are mutually exclusive and defined a priori based on the categorization of the serum antibody levels for 19 periodontal bacteria made by Merchant et al. “Association between Serum Antibodies to Oral Microorganisms and Hyperglycemia in Adults” (2014), using NHANES III data.

BMI = body mass index, CI = confidence interval, NHANES = National Health and Nutrition Examination Survey.

* Adjusted for age, sex, race, education, income, smoking, BMI, alcohol.

† Orange-Blue cluster: *E nodatum*, *A naeslundii*.

‡ Yellow-Orange cluster: *S. intermedius*, *S. oralis*, *S.mutans*, *F.nucleatum*, *P. micra*, *C. ochracea*.

§ Red-Green cluster: *T forsythia*, *T denticola*, *A actinomycetemcomitans* mix, *E corrodens*, *S noxia*, *V parvula*, *C. rectus*.

‖ Orange-Red cluster: *P melaninogenica*, *P intermedia*, *P nigrescens*, *P gingivalis mix*.

### 3.3. Association between clinical periodontal measurements and Vitamin D deficiency

**Figure 3.**
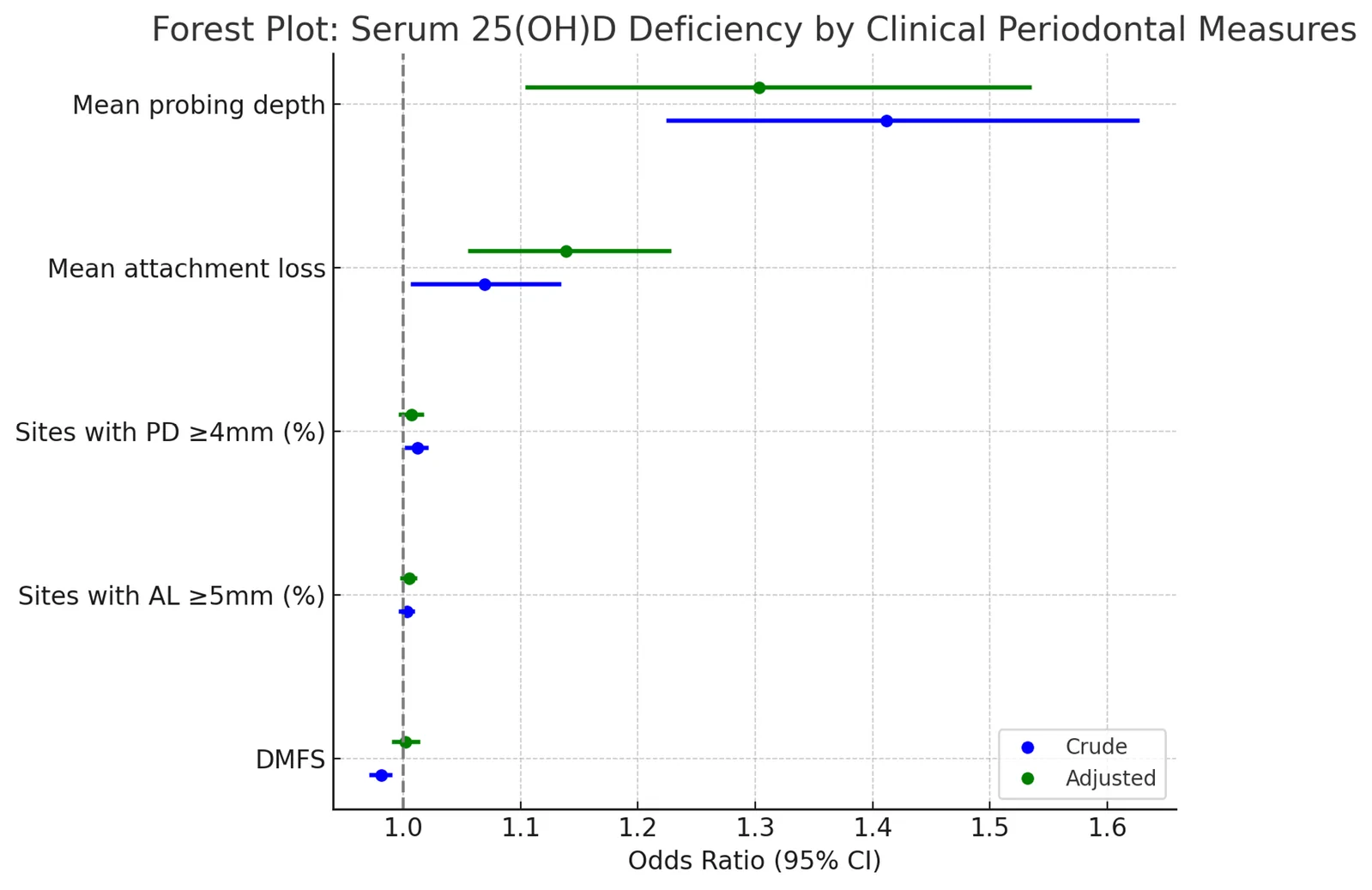
Forest plot of odds ratios (OR) and 95% confidence intervals (CI) for prevalent serum 25-hydroxyvitamin D (25[OH]D) deficiency according to clinical periodontal measures, including mean probing depth (PD), mean attachment loss (AL), percentages of sites with probing depths ≥4 mm and attachment loss ≥5 mm, and decayed, missing, and filled surfaces (DMFS), among participants in NHANES III. NHANES = Third National Health and Nutrition Examination Survey.

Without adjusting for covariates, each 1-unit increase in mean probing depth was associated with a 41.2% increase in the prevalence of Vitamin D deficiency (OR [95% CI] = 1.412 [1.226, 1.626]; *P* < .001) (Fig. 3) (Table 3). Similarly, mean attachment loss was associated with a 6.9% increase in prevalence (OR [95% CI] = 1.069 [1.008, 1.133]; *P* < .05) (Fig. 3) (Table 3). Additionally, for each 1% increase in the percentage of sites with probing depths ≥4 mm, the prevalence increased by 1.2% (OR [95% CI] = 1.012 [1.003, 1.020]; *P* < .01) (Fig. 3) (Table 3). In contrast, DMFS was inversely associated with deficiency in the crude model (OR [95% CI] = 0.981 [0.973, 0.989]; *P* < .001) (Fig. 3) (Table 3). After adjusting for age, sex, education, smoking, alcohol intake, and BMI, the association remained significant for both mean probing depth (OR [95% CI] = 1.303 [1.106, 1.534]; *P* < .01) and mean attachment loss (OR [95% CI] = 1.139 [1.057, 1.227]; *P* < .001) (Fig. 3) (Table 3). However, no statistically significant associations were observed for the percentage of sites with probing depths ≥4 mm, percentage of sites with attachment loss ≥5 mm, or DMFS (all *P* -values > .05) (Fig. 3) (Table 3).

**Table 3:** Odds ratio (95% CI) for prevalent Vitamin D deficiency according to clinical periodontal measures among participants in the third National Health and Nutrition Examination Survey (NHANES III) 1988–1992. (N = 6211).

Clinical periodontal measures	Crude Vitamin D deficiency (5146/6211)	*P*-value	Adjusted* vitamin D deficiency (5146/6211)	*P*-value
Mean probing depth	1.412 (1.226, 1.626)	<.001	1.303 (1.106, 1.534)	<.01
Mean attachment loss	1.069 (1.008, 1.133)	<.05	1.139 (1.057, 1.227)	<.001
Percentage of site with probing depths ≥ 4mm (%)	1.012 (1.003, 1.020)	<.01	1.007 (0.998, 1.016)	.128
Percentage of site with attachment loss ≥ 5mm (%)	1.003 (0.998, 1.008)	.22	1.005 (0.999, 1.010)	.08
DMFS	0.981 (0.973, 0.989)	<.001	1.002 (0.992, 1.013)	.69

Mean probing depths and attachment loss sites are based on the mesiobuccal and midbuccal sites of all fully erupted teeth, excluding 3^rd^ molars, in 2 randomly selected quadrants.

Odds ratios are for either a 1 mm increase in probing depth or attachment loss; either a 1 % increase in the percentage of site with probing depth ≥ 4mm (%) or attachment loss ≥ 5mm (%); or a 1-unit increase in DMFS.

BMI = body mass index, CI = confidence interval, DMFS = decayed, missing, and filled surfaces, NHANES = National Health and Nutrition Examination Survey.

* Adjusted for age, sex, race, education, income, smoking, BMI, and alcohol.

### 3.4. Association between the IgG levels of the nineteen periodontal bacteria and vitamin D deficiency

**Figure 4.**
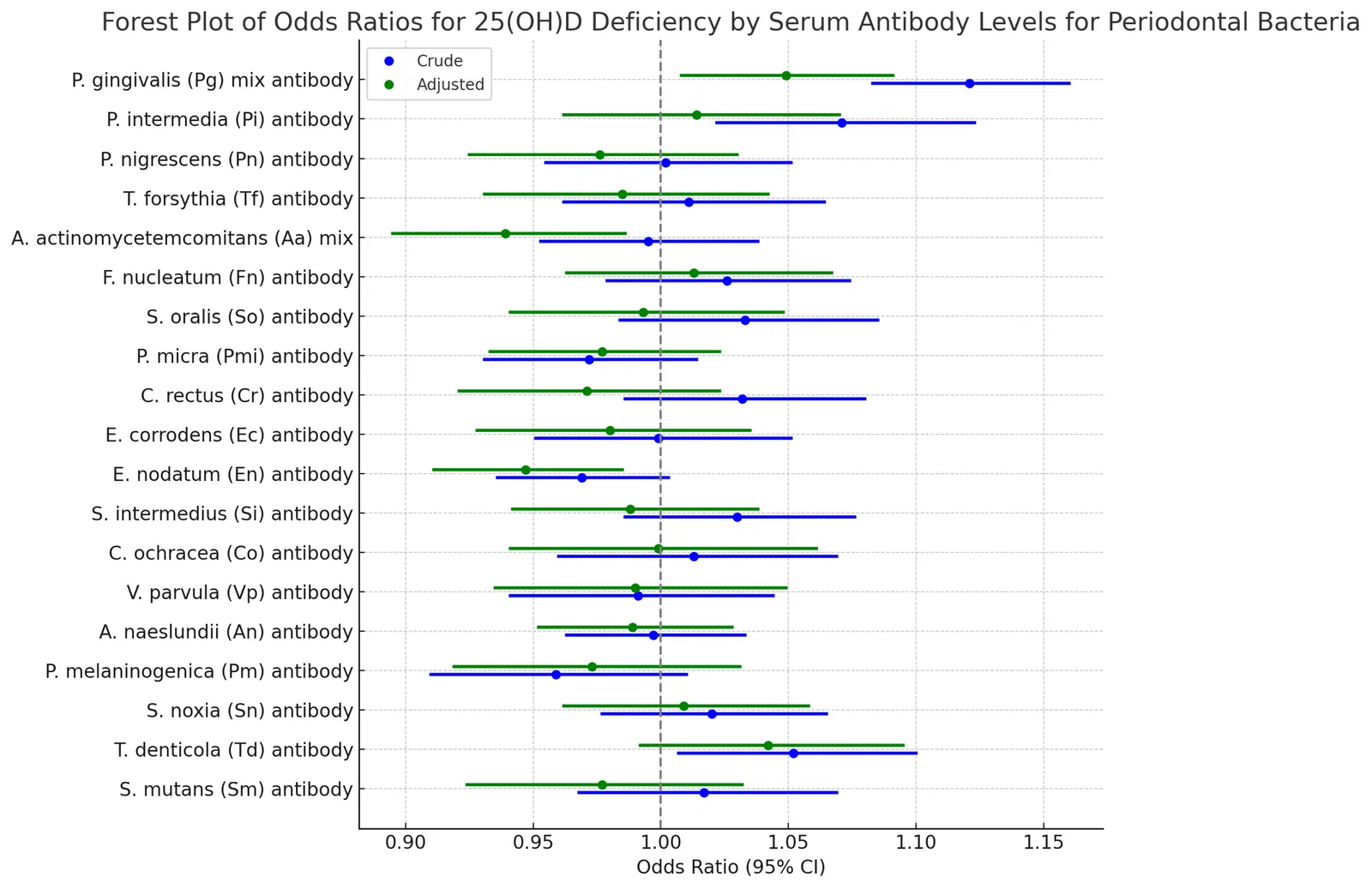
Forest plot of odds ratios (OR) and 95% confidence intervals (CI) for prevalent serum 25-hydroxyvitamin D (25[OH]D) deficiency according to serum IgG antibody levels against 19 periodontal bacteria among participants in NHANES III. NHANES = Third National Health and Nutrition Examination Survey.

Without adjusting for covariates, each 1-unit increase in *P gingivalis* (Pg) mix antibody was associated with a 12.1% increase in the prevalence of Vitamin D deficiency (OR [95% CI] = 1.121 [1.083, 1.160]; *P* < .001) (Fig. 4) (Table 4). Similarly, higher antibody levels of *P. intermedia* (Pi) (OR = 1.071 [1.022, 1.123]; *P* < .01) and *T. denticola* (Td) (OR = 1.052 [1.007, 1.100]; *P* < .05) were significantly associated with increased odds of Vitamin D deficiency (Fig. 4) (Table 4). After adjusting for age, sex, race, education, income, smoking, BMI, and alcohol intake, only *P gingivalis* (Pg) mix antibody remained significantly associated with Vitamin D deficiency (OR [95% CI] = 1.049 [1.008, 1.091]; *P* < .05) (Fig. 4) (Table 4). Additionally, *A actinomycetemcomitans* (Aa) mix and *E nodatum* (En) antibodies were negatively associated with Vitamin D deficiency in the adjusted model (Aa: OR = 0.939 [0.895, 0.986]; *P* < .05; En: OR = 0.947 [0.911, 0.985]; *P* < .01) (Fig. 4) (Table 4). No other significant associations were observed between antibody levels of the remaining 16 periodontal bacteria and Vitamin D deficiency (all *P* -values > .05) (Fig. 4) (Table 4).

**Table 4:** Odds ratio (95% CI) for prevalent vitamin D deficiency according to serum antibody levels for 19 periodontal bacteria among participants in the third National Health and Nutrition Examination Survey (NHANES III) 1988–1992. (N = 6211).

Periodontal bacteria antibody levels	Crude vitamin D deficiency (5146/6211)	*P*-value	Adjusted* vitamin D deficiency (5146/6211)	*P*-value
*P. gingivalis* (Pg) mix antibody	1.121 (1.083, 1.160)	<.001	1.049 (1.008, 1.091)	<.05
*P. intermedia* (Pi) antibody	1.071 (1.022, 1.123)	<.01	1.014 (0.962, 1.070)	.599
*P. nigrescens* (Pn) antibody	1.002 (0.955, 1.051)	.929	0.976 (0.925, 1.030)	.378
*T. forsythia* (Tf) antibody	1.011 (0.962, 1.064)	.658	0.985 (0.931, 1.042)	.603
*A. actinomycetemcomitans* (Aa) mix	0.995 (0.953, 1.038)	.810	0.939 (0.895, 0.986)	<.05
*F. nucleatum* (Fn) antibody	1.026 (0.979, 1.074)	.281	1.013 (0.963, 1.067)	.612
*S. oralis* (So) antibody	1.033 (0.984, 1.085)	.192	0.993 (0.941, 1.048)	.802
*P. micra* (Pmi) antibody	0.972 (0.931, 1.014)	.181	0.977 (0.933, 1.023)	.317
*C. rectus* (Cr) antibody	1.032 (0.986, 1.080)	.181	0.971 (0.921, 1.023)	.266
*E. corrodens* (Ec) antibody	0.999 (0.951, 1.051)	.993	0.980 (0.928, 1.035)	.465
*E nodatum* (En) antibody	0.969 (0.936, 1.003)	.070	0.947 (0.911, 0.985)	<.01
*S intermedius* (Si) antibody	1.030 (0.986, 1.076)	.186	0.988 (0.942, 1.038)	.637
*C ochracea* (Co) antibody	1.013 (0.960, 1.069)	.634	0.999 (0.941, 1.061)	.971
*V parvula* (Vp) antibody	0.991 (0.941, 1.044)	.737	0.990 (0.935, 1.049)	.741
*A naeslundii* (An) antibody	0.997 (0.963, 1.033)	.885	0.989 (0.952, 1.028)	.584
*P melaninogenica* (Pm) antibody	0.959 (0.910, 1.010)	.110	0.973 (0.919, 1.031)	.361
*S noxia* (Sn) antibody	1.020 (0.977, 1.065)	.376	1.009 (0.962, 1.058)	.722
*T denticola* (Td) antibody	1.052 (1.007, 1.100)	<.05	1.042 (0.992, 1.095)	.099
*S mutans* (Sm) antibody	1.017 (0.968, 1.069)	.507	0.977 (0.924, 1.032)	.404

BMI = body mass index, CI = confidence interval, NHANES = National Health and Nutrition Examination Survey.

* Adjusted for age, sex, race, education, income, smoking, BMI, and alcohol.

## 4. Discussion

According to our research, those who had a Vitamin D deficiency were typically younger, primarily female, non-Hispanic Black, nondrinking, nonsmoking, and obese. In comparison to their healthy peers, they also tended to have lower household incomes and worse dental and periodontal health. These socioeconomic and demographic differences are consistent with previous research, which emphasizes the multidimensional nature of risk factors for Vitamin D deficiency, which include lifestyle, health-related behaviors, and demographic traits.^[6,30,31]^

In the unadjusted analysis, each 1-unit increase in the Orange-Red cluster score was associated with increased prevalence of Vitamin D deficiency. The orange-red cluster includes *P gingivalis* and, compared to the other 3 clusters, primarily represents characteristics of periodontal disease.^[32,33]^ However, after adjusting for covariates, this association lost statistical significance, suggesting a potentially weak relationship that may be confounded by multiple factors.

Subsequently, we also found that Vitamin D deficiency was significantly associated with poorer periodontal parameters. Specifically, greater mean probing depth and mean attachment loss remained independently associated with increased odds of vitamin D deficiency after adjusting for confounding factors. These findings are consistent with previous clinical studies suggesting a potential inverse relationship between vitamin D status and periodontal inflammation.^[13,34]^ Mechanistically, vitamin D exerts its effects by binding to the vitamin D receptor (VDR), a member of the nuclear receptor superfamily and a ligand-activated transcriptional regulator.^[35]^ Vitamin D can reduce the activation of the NALP3 inflammasome via VDR-mediated pathways, leading to decreased IL-1β production,^[36]^ which in turn contributes to the alleviation of periodontal inflammation.^[37]^ Additionally, vitamin D has demonstrated inhibitory effects on common oral pathogens, which may represent another important pathway by which it mitigates periodontitis. For example, it has been reported that vitamin D can upregulate human β-defensin 3 in human gingival epithelial cells and human periodontal ligament (HPL) cells, thereby suppressing the adhesion and infection of *P gingivalis*.^[18]^ Another study suggested that vitamin D promotes the co-localization of *P gingivalis* with autophagosomes and lysosomes, facilitating its degradation through autophagy.^[17]^ These findings also suggest that the protective effect of vitamin D on periodontal disease may not be limited to its regulation of *P gingivalis* alone, but could also involve interactions with other periodontal pathogens.

Therefore, we further analyzed the association between IgG levels against nineteen periodontal bacteria and Vitamin D deficiency. After adjusting for potential confounders: including age, sex, race, education, income, smoking status, BMI, and alcohol intake: we found that only IgG levels against *P gingivalis (Pg) mix* remained significantly positively associated with Vitamin D deficiency (OR [95% CI] = 1.049 [1.008, 1.091]; *P* < .05). This finding supports the hypothesis that vitamin D may be associated with changes in periodontal pathogen levels.^[17,18,38]^ The magnitude of this association, however, was modest, with the CI narrowly above 1.0, suggesting a relatively weak signal. In contrast, antibody levels against *A actinomycetemcomitans* (Aa) mix and *E nodatum* (En) were negatively associated with Vitamin D deficiency (Aa: OR = 0.939 [0.895, 0.986]; *P* < .05; En: OR = 0.947 [0.911, 0.985]; *P* < .01). The negative association between *A actinomycetemcomitans* and vitamin D deficiency is noteworthy, as although *A actinomycetemcomitans* is commonly recognized as a periodontal pathogen; particularly among adolescents with aggressive periodontitis^[39]^: its carriage rate varies significantly across different geographic regions and clinical forms of the disease.^[40]^ Individual susceptibility to colonization also varies,^[41]^ and some researchers have proposed that not all *A actinomycetemcomitans* strains possess the same pathogenic potential.^[42]^ Therefore, if the inverse relationship between Vitamin D deficiency and *A actinomycetemcomitans* is indeed causal, its impact on periodontal disease may be minimal. Moreover, since *E nodatum* is also a well-known periodontal pathogen,^[43]^ and previous studies have suggested that it may be potentially associated with endocrine function and bone metabolism.^[44,45]^ We therefore believe that this inverse relationship suggests that whether 25(OH)D is directly associated with levels of *A actinomycetemcomitans* or *E nodatum remains* to be clarified through future in vivo studies. The effect sizes for both associations were modest (approximately 5%–6% reduction in odds per unit increase), with CIs entirely below 1.0 but close to unity, indicating limited strength of these inverse signals.

This study is not without limitations. On one hand, the inherent nature of a cross-sectional design restricts our ability to determine a causal relationship between antibody titers against periodontal pathogens and vitamin D deficiency. In practice, we can only emphasize the potential associations between specific periodontal pathogens and serum vitamin D deficiency. On the other hand, certain potential confounding factors could not be fully accounted for. Vitamin D status is influenced by multiple modifiable factors beyond those measured in NHANES III, including sunlight exposure, seasonal variation, dietary vitamin D intake (e.g., oily fish, fortified foods), and use of vitamin D-containing supplements. The current analytical cohort lacked detailed data on these factors, which may have introduced residual confounding.^[28]^ Notably, the direction of this potential bias is not easily predictable: individuals with higher sun exposure or supplement use may have better overall health behaviors, potentially attenuating the observed associations toward the null; conversely, if these factors are correlated with both vitamin D status and periodontal pathogen exposure through shared lifestyle pathways (e.g., outdoor activity, health-seeking behavior), the observed effect sizes may be overestimated. Furthermore, the NHANES III data were collected between 1988 and 1994, a period when vitamin D supplementation and fortification practices differed substantially from today, limiting the generalizability of our findings to contemporary populations with different dietary and supplementation patterns. Third, given the exploratory nature of the 19 individual bacterial antibody analyses, we did not apply strict multiple-comparison corrections (e.g., Bonferroni). While this approach reduces the risk of type II errors (false negatives) and allows the detection of novel signals, we acknowledge that it may inflate the type I error rate (false positives). Therefore, the significant associations observed for individual bacteria (*P gingivalis, A actinomycetemcomitans*, and *E nodatum*) should be interpreted as hypothesis-generating rather than confirmatory, and warrant validation in future independent cohorts with prespecified correction strategies. Nevertheless, our study also has several strengths. First, it examined the association between serum vitamin D deficiency and antibody levels against 19 periodontal pathogens, offering a more comprehensive perspective compared to previous studies that focused on a single pathogen, such as *P gingivalis*. Second, antibody titers against periodontal pathogens can remain elevated in the bloodstream for 15 to 30 years following initial infection.^[22,23]^ Compared to direct detection of pathogens in the oral environment, this serological approach may better reflect the historical exposure to periodontal infection and its relationship with vitamin D deficiency in otherwise healthy individuals. Lastly, given the large and nationally representative sample used in this study, the likelihood of false-negative results was substantially reduced.

## 5. Conclusion

In this cross-sectional study, we found no evidence of an association between antibody clusters and vitamin D insufficiency in the nationally representative NHANES III population. However, specific bacteria (*P gingivalis*, *A actinomycetemcomitans*, and *E nodatum*) were associated with vitamin D status. Given the cross-sectional design, these findings demonstrate associations rather than causality, and prospective studies are warranted to determine temporality.

## Author contributions

**Conceptualization:** Li Tan, Si-Qun Xu.

**Data curation:** Li Tan.

**Formal analysis:** Li Tan.

**Investigation:** Li Tan.

**Resources:** Li Tan.

**Software:** Li Tan.

**Supervision:** Li Tan, Si-Qun Xu.

**Visualization:** Li Tan, Si-Qun Xu.

**Project administration:** Si-Qun Xu.

**Validation:** Si-Qun Xu.

**Writing – original draft:** Li Tan.

**Writing – review & editing:** Li Tan, Si-Qun Xu.
